# Sex-Differential Selection and the Evolution of X Inactivation Strategies

**DOI:** 10.1371/journal.pgen.1003440

**Published:** 2013-04-18

**Authors:** Tim Connallon, Andrew G. Clark

**Affiliations:** Department of Molecular Biology and Genetics, Cornell University, Ithaca, New York, United States of America; University of Otago, New Zealand

## Abstract

X inactivation—the transcriptional silencing of one X chromosome copy per female somatic cell—is universal among therian mammals, yet the choice of which X to silence exhibits considerable variation among species. X inactivation strategies can range from strict paternally inherited X inactivation (PXI), which renders females haploid for all maternally inherited alleles, to unbiased random X inactivation (RXI), which equalizes expression of maternally and paternally inherited alleles in each female tissue. However, the underlying evolutionary processes that might account for this observed diversity of X inactivation strategies remain unclear. We present a theoretical population genetic analysis of X inactivation evolution and specifically consider how conditions of dominance, linkage, recombination, and sex-differential selection each influence evolutionary trajectories of X inactivation. The results indicate that a single, critical interaction between allelic dominance and sex-differential selection can select for a broad and continuous range of X inactivation strategies, including unequal rates of inactivation between maternally and paternally inherited X chromosomes. RXI is favored over complete PXI as long as alleles deleterious to female fitness are sufficiently recessive, and the criteria for RXI evolution is considerably more restrictive when fitness variation is sexually antagonistic (*i.e.*, alleles deleterious to females are beneficial to males) relative to variation that is deleterious to both sexes. Evolutionary transitions from PXI to RXI also generally increase mean relative female fitness at the expense of decreased male fitness. These results provide a theoretical framework for predicting and interpreting the evolution of chromosome-wide expression of X-linked genes and lead to several useful predictions that could motivate future studies of allele-specific gene expression variation.

## Author Summary

With the exception of its most primitive members, mammal species practice X inactivation, where one copy of each X chromosome pair is silenced in each cell of the female body. The particular copy of the X that is silenced nevertheless shows considerable variability among species, and the evolutionary causes for this variability remain unclear. Here, we show that X inactivation strategies are likely to evolve in response to the sex-differential fitness properties of X-linked genetic variation. Genetic variation with similar effects on male and female fitness will generally favor the evolution of random X inactivation, potentially including preferential inactivation of the maternally inherited X chromosome. Variation with opposing fitness effects in each sex (“sexually antagonistic” variation, which includes mutations that both decrease female fitness and enhance male fitness) selects for preferential or complete inactivation of the paternally inherited X. Paternally biased X inactivation patterns appear to be common in nature, which suggests that sexually antagonistic genetic variation might be an important factor underlying the evolution of X inactivation. The theory provides a conceptual framework for understanding the evolution of X inactivation strategies and generates several novel predictions that may soon be tested with modern genome sequencing technologies.

## Introduction

Mammalian females transcriptionally silence one of their two X chromosomes within each somatic cell – a process called X inactivation [1], [2]. The basic phenomenon of X inactivation occurs in all therian (non egg-laying) mammals studied to date, yet the specific X chromosome silenced exhibits considerable diversity among species. At one extreme, typical of marsupials, the paternally inherited X is universally silenced and the maternally inherited X is ubiquitously expressed ([3], [4]; hereafter referred to as paternal X inactivation or PXI). In contrast, placental mammals practice random X inactivation (RXI): each somatic cell may express either the maternally or the paternally inherited X (the other X is silenced), and female bodies are composed of a mosaic of cells that individually express one of the two X chromosome copies [5]. While RXI is generally thought to be unbiased – with each cell having an equal probability of expressing either of the two X chromosomes – recent data reveal quantitatively biased inactivation patterns in at least some placental mammal species, *i.e.*: differential silencing of maternally and paternally derived X chromosomes [6], [7]. Several marsupial studies similarly find evidence for partial expression of the paternally derived X, suggesting additional species-specificity of X inactivation rules (reviewed in [2], [8]).

The selective processes that might account for this observed diversity remain unclear. A leading hypothesis for the evolution of RXI is that it might be favored if segregating deleterious mutations have recessive or partially recessive fitness effects ([9]–[11]; which, on average, they do [12]–[15]). The logic underlying this hypothesis is straightforward. Females that uniformly silence a particular copy of the X (*e.g.*, the paternally inherited copy under PXI) will be effectively haploid, and suffer the full fitness costs of mutations carried on their expressed X chromosome. RXI generates an expression pattern that is more similar to diploidy, and can potentially mask the fitness costs of carrying deleterious alleles.

While the masking hypothesis for the evolutionary origins of RXI is plausible (*e.g.*, [1], [16]–[20]), its feasibility should be investigated in a formal population genetic model. Models of a similar evolutionary scenario, the evolution of haploid versus diploid life cycles (*e.g.*, [21]–[25]), indicate that selection for masking of deleterious mutations favors the evolution of diploidy, if mutations are sufficiently recessive relative to the population's recombination rate ([26]–[30]; however, selection to mask somatic mutations eliminates constraints imposed by tight linkage [31]). However, these models do not incorporate the unique properties of sex-differential selection and inheritance that govern X chromosome evolution [32], so it remains unclear whether their conclusions apply to the case of RXI.

Sex differences in selection – where the fitness effects of single mutations differ in magnitude or direction between males and females – likely influence large fractions of animal genomes [33]–[36], which can have two potential consequences for the evolutionary diversification of X inactivation strategies. Stronger selection against deleterious alleles in males compared to females should decrease the average proportion of deleterious alleles carried on each paternally derived X (*e.g.*, [10], [33]), and thereby favor expressing the paternally inherited X. “Sexually antagonistic alleles” – alleles that increase fitness when present in one sex, but decrease fitness in the other sex [37], [38] – should have the opposite effect on X inactivation. Alleles benefiting males and costly to females experience higher probabilities of paternal transmission (*e.g.*, [39], [40]), which could generate selection to preferentially inactivate, or even ubiquitously silence, paternally inherited X chromosomes. Several models have examined how sexually antagonistic selection might favor the evolution genomic imprinting, which similarly involves the unequal expression of maternally and paternally inherited gene copies [40]–[44]. However, the effect of sexually antagonistic fitness variation on X inactivation evolution has yet to be addressed.

It is currently unclear how the population genetic parameters of dominance, sex-differential selection, and linkage and recombination might jointly influence the evolution of X inactivation strategies. We therefore developed a mathematical model of X inactivation evolution, and used this model to identify biological conditions that favor the evolution of different X inactivation states. We first consider the dichotomous case of RXI versus PXI, describe the conditions facilitating evolutionary transitions to RXI, and characterize the consequences of such transitions for mean fitness of males and females. Overall, selection on mammalian X inactivation strategies is primarily mediated by the interaction between dominance and sex-differential selection, and the situation differs considerably from scenarios that favor the evolution of diploidy. We also examine whether sex-differential selection might favor the evolution of biased X inactivation strategies (*i.e.*, quantitatively unequal expression of maternally versus paternally derived X chromosomes) and predict the magnitude of biases likely to evolve. Our results suggest that conditions for evolving biased inactivation patterns are extremely permissive.

## Results

### Development of the model

We focus on the simplest and most analytically tractable model that simultaneously incorporates genetic linkage, variation for fitness, and variation in the form of X inactivation. Our model follows the evolution of two bi-allelic loci. Locus *A* (the “fitness locus”) is X-linked and carries *A*_1_ and *A*_2_ alleles, which directly influence male and female fitness. Locus *B* (the “modifier locus”) carries *B*_1_ and *B*_2_ alleles, which influence the X inactivation rule in females within the population. The genotype at locus *B* can influence female fitness through its effect on the relative expression of *A*_1_ versus *A*_2_ alleles in heterozygotes. Variation at the *B* locus has no other phenotypic effect in males or females and is therefore neutral in males. We consider two scenarios of linkage for the *B* locus. Under X-linkage, *A* and *B* are physically linked, and recombine at a rate *r*, per female meiosis (the X does not recombine in males, which have only one X chromosome copy). When *B* is on an autosome, alleles at *A* and *B* loci segregate independently during meiosis.

To model the evolution of X inactivation, we begin with the *B* locus initially fixed for allele *B*_1_, and the *A* locus at polymorphic equilibrium given *B*_1_ fixed. We then characterize selection on and evolution of a novel *B*_2_ allele that changes a female carrier's X inactivation system. The population is assumed to be sufficiently large that genetic drift can be ignored, and each generation is discrete. The life cycle during each generation follows the order of birth, selection, recombination, mutation, random mating and syngamy. Our approach bears many similarities to models for the evolution of ploidy cycles (*e.g.*, [26], [45] chapter 8 of [46]), and for the evolution of sexually dimorphic genomic imprinting [40], [44], with which we draw contrasts. Generalized two-locus recursions (see Methods) include sixteen different female genotypes (when allowing for parent-of-origin effects) and at least four male genotypes (four for the X-linked modifier model; eight for the autosomal modifier model). To reduce the enormous range of possible fitness parameterizations and initial population conditions, we focus our attention on a subset of idealized and biologically relevant population genetic scenarios in the following analyses.

We consider two basic forms of fitness variation at the *A* locus (Table 1): (1) deleterious alleles maintained at a balance between recurrent mutation and purifying selection; and (2) sexually antagonistic alleles stably maintained as balanced polymorphisms. In both scenarios, the female-deleterious allele is denoted by *A*_1_, *i.e.* female fitness is highest in *A*_2_ homozygotes, and fitness of heterozygous females is assumed to be intermediate to the two homozygous genotypes [formally, *w*(*A*_1_ *A*_1_) = 1−*s_f_*≤*w*(*A*_1_ *A*_2_), *w*(*A*_2_ *A*_1_)≤*w*(*A*_2_ *A*_2_) = 1, where *w*(*A_i_A_j_*) is the fitness of a female with genotype *A_i_A_j_*, and *s_f_* describes the fitness cost to females of being homozygous or haploid for the *A*_1_ allele: 1>*s_f_*>0]. When *A*_1_ is also deleterious to males, it will be maintained at mutation-selection balance (*s_m_* is the fitness cost to males of carrying an *A_1_* allele; 1>*s_m_*>0; Table 1). We also consider genetic polymorphism maintained by sexual antagonism, where *A*_2_ is the deleterious allele for males (here, *t_m_* is the fitness cost to males of carrying an *A*_2_ allele; 1>*t_m_*>0; see Table 1).

**Table 1:** Fitnesses and frequencies of genotypes at the *A* locus.^1^

	Females			
Genotype:	*A*_1_ *A*_1_	*A*_1_ *A*_2_	*A*_2_ *A*_1_	*A*_2_ *A*_2_
Frequency in zygotes:	*q_m_q_f_*	*q_f_*(1−*q_m_*)	*q_m_*(1−*q_f_*)	(1−*q_f_*)(1−*q_m_*)
Fitness:	1−*s_f_*	1−*s_f_h_mat_*	1−*s_f_h_pat_*	1

	Males	
Genotype:	*A*_1_	*A*_2_
Frequency in zygotes:	*q_f_*	1−*q_f_*
Fitness (*A*_1_ deleterious):	1−*s_m_*	1
Fitness (*A*_2_ deleterious):	1	1−*t_m_*

1 The maternally inherited allele is listed first and the paternally inherited allele is listed second; frequencies in gametes are *q_f_* = [*A*_1_] in eggs and *q_m_* = [*A*_1_] in sperm; 0<*s_f_*, *s_m_*, *t_m_*, *h_mat_*, *h_pat_*<1.

In an ancestral population fixed for *B*_1_, female somatic cell lineages are assumed to silence the paternally inherited X with probability *ξ*_11_ and silence the maternal X with probability 1−*ξ*_11_ (0≤*ξ*_11_≤1). Under unbiased RXI (where *ξ*_11_ = ½), heterozygous females have fitness *w*(*A*_1_ *A*_2_) = *w*(*A*_2_ *A*_1_) = 1−*s_f_h*, where *h* represents the degree of masking of the *A*_1_ allele (0<*h*<1). Thus, *h* is a scaling factor that is analogous to the dominance coefficient of standard population genetic models. Use of the terms “dominance” and “recessivity”, applied to species with RXI, has been questioned because individual cells lack bi-allelic expression [47]. However, the dominance coefficient remains useful as a population genetic parameter, and simply quantifies the relative fitness of heterozygous versus homozygous genotypes when heterozygotes practice an unbiased RXI rule. Partial masking is clearly relevant for many X-linked disorders, which tend to be less penetrant and less severe in females than males [47].

**Figure 1:**
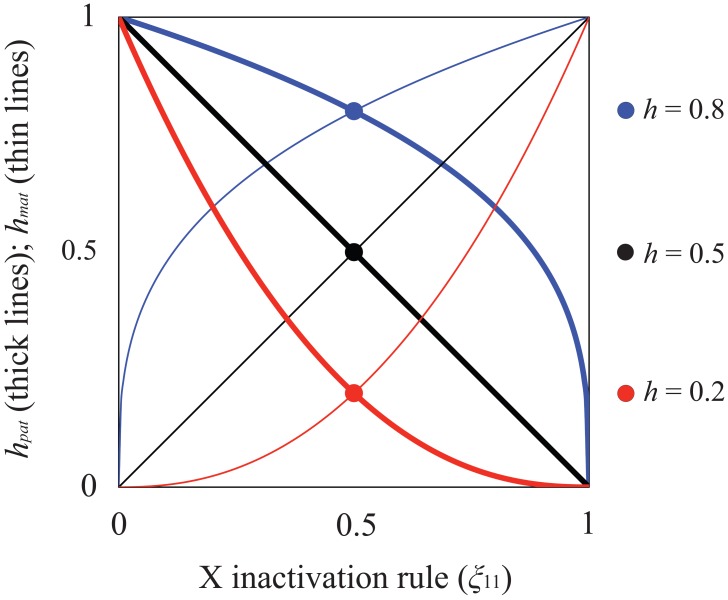
Relationship between X inactivation rule and parent-of-origin dominance coefficients (***h_mat_*** **,** ***h_pat_*** **; see** **Table 1** **).** Results are based on the power function for female fitness, *w*(*x*) = 1−*x^k^s_f_*, where *x* represents the proportion of female cells expressing the *A*_1_ allele, *s_f_* is the haploid or homozygous selection coefficient, *h* is the degree of masking (equivalent to a dominance coefficient of *A*_1_) in individuals practicing unbiased RXI (*ξ*_11_ = ½), and *k* = −ln(2)/ln(*h*). For additional details, see the main text. The figure is modified from, and inspired by, Figure 1a of [50].

When X inactivation is biased (*ξ*_11_≠½), fitness is function of *h* (as defined above) and parent-of-origin effects generated by the X inactivation rule. Assuming that female fitness decreases monotonically with the proportion of cells expressing the *A*_1_ allele, we can describe it using a generalized power function, *w*(*x*) = 1−*x^k^s_f_*, where *x* represents the proportion of cells expressing the *A*_1_ allele, and *k* is a positive constant that describes the specific shape of the fitness decline associated with *A_1_* expression (such functions are often used in evolutionary theory because of their flexibility; *e.g.*, [48]–[50]). For our purposes, *w*(*x*) has two essential properties. First, fitness approaches unity when *A*_2_ is ubiquitously expressed (*i.e.*, 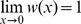), and 1−*s_f_* when *A*_1_ is ubiquitously expressed (*i.e.*, 
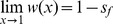
), which makes intuitive sense. Second, *k* can be defined in terms of the dominance coefficient of *A*_1_. When *ξ*_11_ = ½, *x* = *ξ*_11_ = 1−*ξ*_11_; therefore, *h* = 1/2*^k^* and *k* = −ln(*h*)/ln(2). *A*_1_ is partially dominant to *A*_2_ when *h*>½ (*k*<1) and partially recessive when *h*<½ (*k*>1). Consequently, *h_mat_* = *ξ*_11_ *^k^* represents the effective dominance coefficient when *A*_1_ is maternally inherited, and *h_pat_* = (1−*ξ*_11_)*^k^* represents the effective dominance coefficient when *A*_1_ is paternally inherited (Table 1; Figure 1). Nonadditivity of allelic effects (*i.e.*, *h*≠½) can arise when the fitness of an X-linked genotype is not cell-autonomous (*e.g.*, fitness depends on the overall proportion of *A*_1_ versus *A*_2_ expression in female bodies; [43], [51]). With cell-autonomous effects, we can model total fitness as the mean fitness per cell [43], such that *w*(*A*_1_ *A*_2_) = 1−*ξ*_11_ *s_f_* and *w*(*A*_2_ *A*_1_) = 1−(1−*ξ*_11_)*s_f_*, which represents a special case of the generalized power function (*i.e.*, *k* = 1 and *h* = ½).

Given the outlined assumptions, we obtain the following equilibria with respect to the X-linked fitness-determining locus. When *A*_1_ is deleterious to both sexes, its equilibrium frequencies at mutation-selection balance (in females and males, respectively) are:
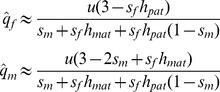
(1)where *u* is the mutation rate to *A*_1_, *h_mat_* = *ξ*_11_ *^k^*, and *h_pat_* = (1−*ξ*_11_)*^k^*, and *k* = −ln(*h*)/ln(2). Eq. (1) was previously derived in [52] (see Text S1). When *A*_1_ is sexually antagonistic (*i.e.*, deleterious to females but beneficial to males), and with balancing selection acting at the *A* locus, the equilibrium frequencies of *A*_1_ are:
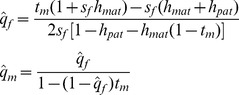
(2)versions of which have been derived in several previous studies [43], [52]–[54]. The balancing selection criteria for sexually antagonistic polymorphism are provided (Text S1; for additional results and discussion, see [43], [52], [54]–[56]).

### Evolution of random X inactivation

PXI is thought to represent the ancestral X inactivation state, from which RXI evolved [1], [57]. We therefore sought to define the population genetic conditions in which a rare *B*_2_ allele that causes unbiased RXI (where *ξ*_12_ = ½ represents the X inactivation strategy played by individuals heterozygous at the modifier locus) will invade a population that is initially fixed for the PXI strategy (*ξ*_11_ = 1).

#### Mutation-selection balance

**Figure 2:**
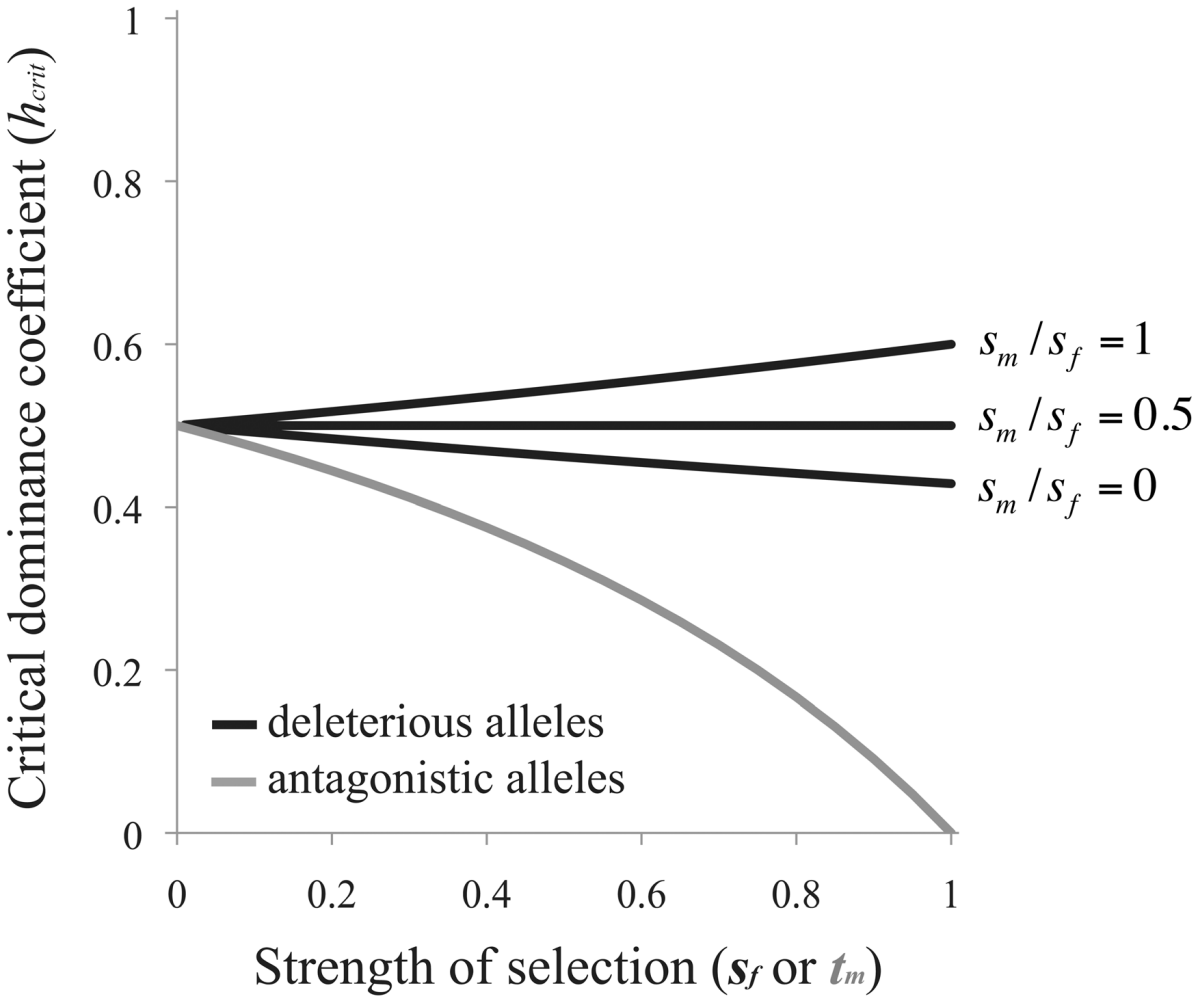
Criteria for the evolution of RXI. Black curves are based on eq. (3) for the mutation-selection balance model of genetic variation (in which case, the *x*-axis refers to the female selection coefficient, *s_f_*). The gray curve is based on eq. (4) for sexually antagonistic alleles maintained by balancing selection (here, the *x*-axis refers to the male selection coefficient: *t_m_*). The area above each curve represents parameter space where unbiased RXI is not favored over PXI. RXI is favored under the complementary parameter space below each curve.

To examine whether segregating recessive deleterious mutations might be sufficiently masked by RXI to render such a strategy favorable to PXI ([10], [11]; see above), we performed a linear stability analysis using our general recursions (see Methods), and evaluated at the equilibrium with *B*_1_ fixed in the population and *A*_1_ at mutation-selection balance. As predicted, selection favors invasion of a modifier allele (*B*_2_) causing RXI when the deleterious allele is sufficiently masked in heterozygotes (*i.e.*, *h* is sufficiently small). Assuming weak mutation at locus *A* (0<*u*≪1), the critical dominance coefficient under RXI is approximately:
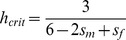
(3)Selection favors the evolution of RXI when *h*<*h_crit_* (Figure 2). Note that Eq. (3) is independent of the recombination rate between the *A* and *B* loci. This result applies equally to scenarios of X and autosomal linkage of the modifier locus.

When selection is weak (*s_m_*, *s_f_*→0), deleterious alleles need only be partially masked (*h*<½) for RXI to be favored. Increasing the strength of purifying selection alters *h_crit_*, with stronger selection in males than females (large *s_m_*/*s_f_* ratios) expanding the parameter space in which RXI evolves, and female-biased selection decreasing it (small *s_m_*/*s_f_* ratios). This effect of differing selection strengths between the sexes has two contributing causes. First, expression of the paternally inherited X (as opposed to the maternal X) is favored because the frequency of deleterious mutations is lower on the paternal X. Second, purifying selection, primarily in males, limits the buildup of linkage disequilibrium (LD) between *B*_2_ alleles and deleterious mutations, which can also prevent the invasion of *B*_2_ alleles. This LD is generated from the epistatic interaction for female fitness between *A* and *B* locus genotypes, and there is no such epistasis in males. Limited evolution of LD expands the parameter space of dominance that permits the evolution of RXI.

#### Sexually antagonistic variation

Sexually antagonistic variation for fitness or its components has been detected in a variety of natural and experimental populations (*e.g.*, [58]–[66]). Sexually antagonistic alleles polymorphic at an X-linked locus could potentially exert selection on X inactivation strategies. Our linear stability analysis of a population fixed for *B*_1_, with a deterministic balanced polymorphism for male-beneficial and female-deleterious alleles (eq. (2), with *ξ*_11_ = 1), shows that RXI will evolve when the dominance coefficient for the female-deleterious allele falls below the following threshold:
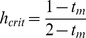
(4)which is again independent of the linkage relationships between loci. Under weak selection (*t_m_*→0), female-detrimental alleles need only be partially masked for RXI to be favored, but overall, the conditions are considerably more restrictive than the mutation-selection balance model. Strong sexually antagonistic selection severely reduces the parameter space that favors the evolution of RXI (Figure 2). Thus, RXI can be selected against, despite strong effects of masking female-detriment alleles. This occurs when strong selection in males causes male-benefit/female-deleterious alleles to be disproportionately transmitted by male gametes.

### Changes in mean fitness when RXI evolves

Evolutionary transitions that influence gene ploidy levels (*e.g.*, transitions from haploidy to diploidy) permit deleterious mutations to accumulate within populations, and reduce long-term population fitness (*i.e.*, the new equilibrium fitness [21], [67]; however, epistasis can sometimes render diploidy advantageous [23]), which is why recombination is required for diploidy to evolve [26]–[30]. Under tight linkage, alleles for diploidy are co-transmitted with deleterious mutations, and fitness benefits of masking can be outweighed by the increased burden of linked, deleterious mutations. Recombination decouples such associations, and permits diploidy to evolve, despite the long-term fitness cost. Such interactions between ploidy and recombination have parallels in various other aspects of genome evolution, including the evolution of genomic imprinting [44], [68] and the establishment of gene duplicates [69], [70].

In contrast, the evolution of RXI does not require recombination (as shown above), and this insensitivity to linkage can similarly be considered in light of mean fitness changes that follow an evolutionary transition from PXI to RXI. For the mutation-selection balance model, equilibrium mean male and female fitnesses (respectively) under PXI and unbiased RXI are:
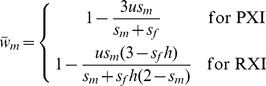
(5a)and
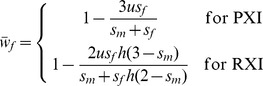
(5b)which each ignore terms of *O*(*u* ^2^). For the parameter space where RXI can evolve [*h*<3/(6−2*s_m_*+*s_f_*); see eq. (3)], mean male fitness is always lower under RXI (as long as *s_m_*>0; otherwise fitness does not change). Mean female fitness becomes higher, as the evolution of RXI shifts some of the burden of purifying selection (removing deleterious mutations) from females to males. The fitness cost to males at the new equilibrium is offset by fitness gains to females, which provides an intuitive explanation as to why selection for RXI is insensitive to linkage. Tightly linked deleterious alleles may hitchhike with a modifier for RXI, yet such associations do not overturn the net benefits of masking.

**Figure 3:**
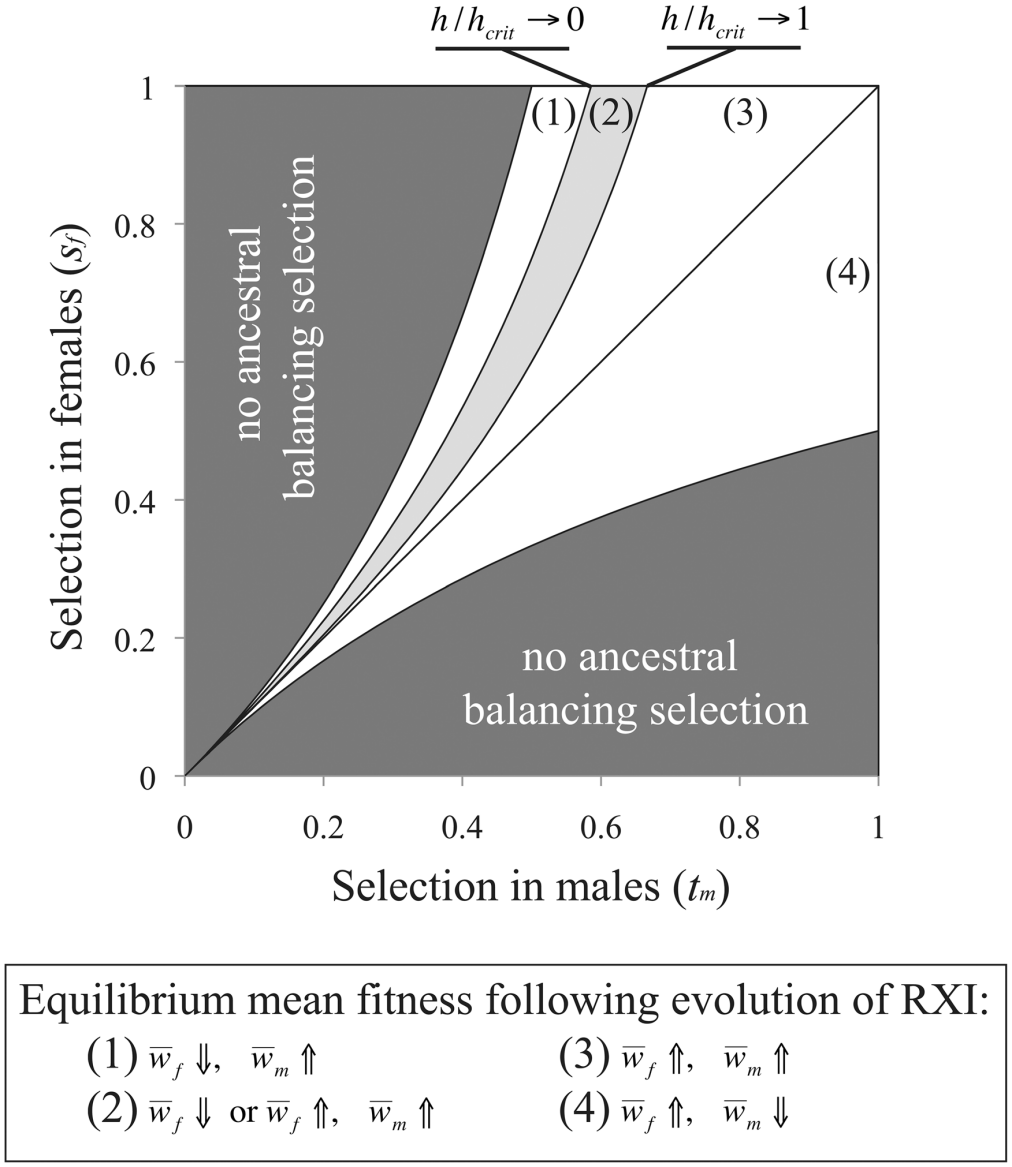
Sexually antagonistic fitness variation and the change in mean fitness following the evolution of RXI. In an ancestral population with PXI, and segregating for a sexually antagonistic balanced polymorphism, unbiased RXI is favored and may evolve when *h*<*h_crit_* = (1−*t_m_*)/(2−*t_m_*). Following such an evolutionary transition, the male-beneficial/female-detrimental allele approaches a new equilibrium frequency, and mean fitness per sex evolves to a new equilibrium.

For the case of an evolutionary transition from PXI to RXI driven by sexually antagonistic polymorphism [in the parameter space where RXI can evolve, *i.e.*: *h*<(1−*t_m_*)/(2−*t_m_*); see eq. (4)], the new equilibrium frequency of the male-beneficial/female-detrimental allele will be higher when *t_m_*<*s_f_*, and lower when *t_m_*>*s_f_*. Mean male fitness is therefore increased after the transition when *s_f_*>*t_m_*, decreased when *s_f_*<*t_m_*, and otherwise remains unchanged. The mean female fitness is increased under a much broader range of conditions, because the masking effect caused by RXI can sometimes offset a higher derived frequency of the female-deleterious allele. The condition necessary for female fitness to be increased following the evolution of RXI is:
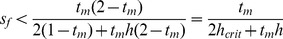
(6)where *h_crit_* = (1−*t_m_*)/(2−*t_m_*). As shown in Figure 3, parameter conditions favoring the evolution of RXI generally lead to an increase in mean female fitness, whereas mean male fitness is increased in exactly half of the relevant parameter space. The new mean fitness is always increased in one sex at least, and in some cases, in both sexes.

For the model of sexually antagonistic genetic variation, our analysis of the change in equilibrium mean fitness follows the evolution of the ancestrally polymorphic locus, and ignores any sexually antagonistic X-linked mutations that might enter the population following the transition to RXI. However, the parameter space that permits an X-linked sexually antagonistic polymorphism is larger under RXI than PXI, within the relevant parameter space where RXI can evolve (PXI can nevertheless be more conducive to polymorphism under alternative dominance parameterizations [52]), and recessive sexually antagonistic alleles that benefit males weakly can more readily be maintained [54], [71]. Therefore, the evolution of RXI could increase the parameter space that permits X-linked sexually antagonistic polymorphism. Antagonistically selected X-linked loci that could not establish balanced polymorphisms in the ancestral population with PXI may do so in an RXI population. In the longer-term, this could increase male and decrease female fitness, contingent on the specific distribution of male and female selection and dominance coefficients among sexually antagonistic mutations.

### The evolution of biased RXI

Thus far we have shown that RXI can be favored by selection if female-detrimental alleles are sufficiently masked when heterozygous. However, selection might not necessarily favor the same rate of inactivation for maternally and paternally derived X chromosomes in females. Sex-differential selection generates allele frequency differences between males and females, which can favor differential expression of genes inherited from opposite-sex parents [40]. With respect to the mammalian X, polymorphism under sex-differential selection might favor the evolution of unequal inactivation rates between paternally and maternally derived X chromosomes; this is conceptually similar to genomic imprinting that involves partial, but unequal, expression of both of the parental gene copies [72]–[79]. Because X inactivation is a female-limited trait, we expect that selection will favor preferential inactivation of the chromosome with a greater probability of carrying female-deleterious alleles.

We tested this intuition by performing an invasion analysis ([46], chapter 12) to determine the evolutionary stability of different X inactivation strategies. To characterize the direction and magnitude of the bias favored by selection, we consider a population initially fixed for an arbitrary inactivation strategy, *ξ*_11_ (0<*ξ*_11_<1), and at equilibrium for fitness variation given this strategy [*e.g.*, eqs. (1–2)], and identify the *ξ*_11_ values that are stable to invasion by an allele that alters the X inactivation ratio of female carriers (*i.e.* the “evolutionarily stable strategies” [80]).

**Figure 4:**
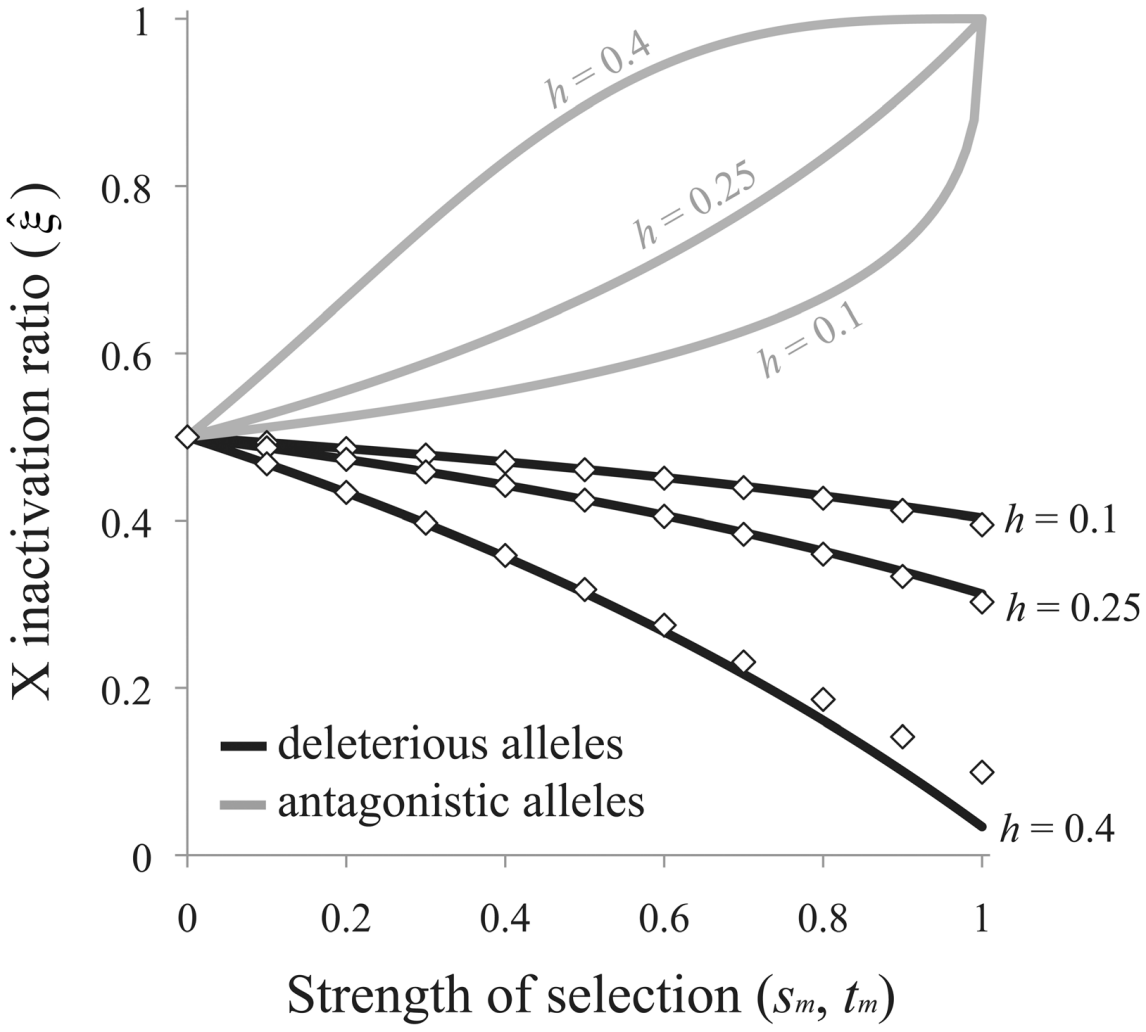
Sex-differential selection favors the evolution of biased RXI. Black curves are based on eq. (8) for the mutation-selection balance model of genetic variation; diamonds are based on numerical evaluation of the more exact eq. (7). Gray curves are based on eq. (9) for sexually antagonistic alleles maintained by balancing selection. Results for the mutation-selection balance case assume equal male and female selection coefficients (*s_m_* = *s_f_*). Biases are further accentuated when *s_m_*>*s_f_*; biases may be dampened or reversed when *s_m_*<*s_f_*.

Under a mutation-selection balance model, the stable paternal X inactivation value (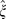) is:
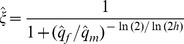
(7)The exponent term [−ln(2)/ln(2*h*)] is positive when *A*_1_ is at least partially recessive (*h*<½), which we assume here and below, and the ratio 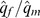 determines the direction of bias favored by selection. Selection favors preferential inactivation of the paternal X when deleterious alleles are more frequently transmitted to progeny through males than females (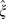
>½ when 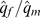
<1), and favors preferential inactivation of the maternal X when deleterious mutations are more frequently transmitted through females (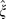
<½ when 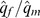
>1). Eq. (7) can be expressed as an explicit function of the selection and dominance coefficients by assuming that *s_f_h*≪1 (this is biologically reasonable given the observed negative association between dominance and effect size of deleterious mutations [12]). The critical ratio becomes:
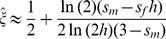
(8)Selection favors preferential inactivation of the maternally derived X when *s_m_*>*s_f_h*, and the degree of bias may be pronounced when deleterious mutations are poorly masked in females (*e.g.*, when *h* is closer to ½ than to zero; Figure 4).

Adopting the same analytical approach for the case of sexually antagonistic fitness variation, the equilibrium X inactivation rule is:
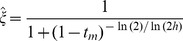
(9)Given the stated parameter constraints (*h*<½; 1>*t_m_*>0), (1−*t_m_*)^−ln(2)/ln(2*h*)^ will always be positive and less than one, and selection always favors preferential inactivation of the paternally inherited X chromosome. The magnitude of this bias increases with *t_m_* and *h*, similar to the deleterious mutation scenario for fitness variation (Figure 4). When either the selection or the dominance coefficient is sufficiently large, both models of fitness variation can favor large X inactivation biases. Some moderate values of *s*, *t* and *h* (to the left of the *x*-axis in Figure 4) could lead to large enough biases – on the order of a few percent or more – to be empirically detectable.

## Discussion

X inactivation in mammals can take a variety of specific forms, ranging from strict PXI, to various forms of RXI [1], [2], [6], [7]. This range of observed diversity is particularly striking, as quantitative analyses of the maternally to paternally derived X inactivation ratio are available for relatively few mammalian species to date (the best data coming from a subset of mouse and human tissues). Modern high-throughput, genome-wide and allele-specific gene expression technologies (such as RNA-seq), can now be used to systematically analyze X inactivation ratios in any number of mammalian species or tissues [6], [81], and this new technology promises to reveal much more diversity in X inactivation patterns.

The range of X inactivation rules employed by different species might usefully be considered within a theoretical framework of sex-differential selection. The population genetic models analyzed here reveal broad opportunities for X inactivation evolution, though it is important to note that model predictions hinge upon the capacity of X inactivation ratios to evolve (*i.e.*, there must be genetic variation for X inactivation rules). There is some evidence for alleles in mice that affect X inactivation choice (reviewed in [82], [83]). Moreover, the observed range of X inactivation patterns between mice, humans, and marsupials (see introduction) imply at least some degree of evolutionary lability for the trait. On the other hand, maternally biased X inactivation has not been observed, and it is unclear whether this reflects under-sampling (the phenomenon may exist, but has yet to be documented), intrinsic genetic constraints (as mentioned), or that biased inactivation of the maternally derived X is generally unfavorable. The evolvability of the X inactivation ratio should be considered an important issue that warrants future study.

### Multiple genes, conflicting patterns of selection, and the evolution of genomic imprinting

For reasons of tractability, and in parallel with theory on the evolution of diploidy (*e.g.*, [26], [45]; chapter 8 of [46]), we have focused our analysis on two-locus population genetics models to characterize how different forms of fitness variation and linkage will influence patterns of selection for different X inactivation rules. However, X inactivation affects the expression of many genes simultaneously, and unique patterns of genetic variation among X-linked loci could individually favor discordant X inactivation strategies. We can gain some insight into scenarios that involve multiple X-linked loci, under the assumption that each polymorphic locus contributes independently to selection on a rare, unlinked modifier allele (*i.e.*, we assume loose linkage and no epistasis between fitness loci; net fitness effects of multi-gene haplotypes could instead apply under tight linkage between fitness loci). Under these conditions, criteria for invasion of a modifier allele will be 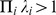, where *λ_i_* is the leading eigenvalue associated with the two-locus system for the modifier locus and the *i*th of *n* X-linked fitness loci (*i* = {1, 2, …, *n*}) [84], [85]. With weak effects of individual fitness loci on the modifier (*i.e.*, *λ_i_*≈1), the selection coefficient associated with a rare modifier allele will be *s_mod_*≈*n*E(*λ_i_*−1), where E(*λ_i_*−1) represents the arithmetic mean, and *λ_i_*−1 represents selection contributed by the *i*th locus (invasion of the modifier allele requires that *s_mod_*>0).

To the extent that X-linked loci segregate for deleterious alleles, and these have similar selection and dominance coefficients, the net effects of multiple X-linked loci on a modifier will be reinforcing, and the strength of selection on the X inactivation strategy will increase with the number of contributing loci (*i.e.*, if E(*λ_i_*−1)≠0, then *s_mod_* scales approximately linearly with *n*, the number of contributing loci; this scaling is in agreement with multilocus models for the evolution of diploidy [28]). Although little available data directly bear upon the sex-specific selection and dominance parameters of mammalian X-linked mutations, data from other systems suggest that most mutations have small, at least partially recessive fitness effects (*e.g.*, [12]–[15], [86]), and are deleterious to both sexes [35], [36]. If X-linked mutations have similarly small selection and dominance coefficients under RXI, then fitness variation due to deleterious alleles might favor the evolution of relatively unbiased RXI rules.

Genes that are polymorphic for sexually antagonistic alleles could exert disproportionately strong influence on the evolutionary trajectories of X inactivation evolution. Sexually antagonistic alleles generate selection of a higher order of magnitude than loci at mutation-selection balance (Figure S1). Consequently, selection due to sexually antagonistic polymorphism may plausibly eclipse the cumulative effects of selection from deleterious alleles. The relative contribution of deleterious versus antagonistically selected alleles to X-linked fitness variation is ultimately an empirical question. To the extent that sexually antagonistic fitness variation is common in mammalian populations (as may indeed be the case; *e.g.*, [60], [64]), selection should favor the evolution of biased inactivation of the paternally inherited X.

Conflicts between different X-linked loci over the optimal ratio of maternal to paternal X inactivation could potentially be resolved by the evolution of genomic imprinting at individual X-linked genes. Previous models have considered two scenarios of sexually antagonistic selection driving the evolution of imprinting (*i.e.*, the partial or complete silencing of a maternally or a paternally inherited copy of a single gene; [72]–[79]). First, when the optimal transcription level of a X-linked gene differs between males and females, imprinting can facilitate sex-specific adaptation by generating sexually dimorphic gene expression [41], [42]. Imprinting of the maternally inherited gene is favored under selection for higher transcription levels in females, whereas paternal imprinting is favored at genes selected for higher transcription in males [41]–[43]. Second, in genes polymorphic for sexually antagonistic alleles, sexually dimorphic imprinting can mitigate fitness costs of inheriting harmful alleles, which are preferentially transmitted from opposite-sex parents [40]. Existing models of this latter scenario consider polymorphism and imprinting at an autosomal gene [40], [44], yet the basic processes should apply to the X (as discussed in [40]) – particularly so because X-linkage promotes allele frequency differences and asymmetrical imprinting effects between males and females [41], [42], [87]. X inactivation and gene-by-gene imprinting may serve as complementary mechanisms for optimizing male and female fitness.

### Evolutionary transitions between RXI and PXI

Evolution of RXI from an ancestral population with PXI should be relatively unconstrained, provided three conditions are met: (*i*) there is genetic variation for RXI (*i.e.*, it is evolvable; see above); (*ii*) X-linked fitness variation is largely caused by segregating deleterious mutations; and (*iii*) the fitness costs of these mutations are at least partially masked under RXI (*i.e.*, *h*<½, as seems likely). The availability of mutations to RXI could potentially constrain the convergent evolution of RXI in marsupials [18]. Sexually antagonistic X-linked fitness variability, if common within marsupials, could also promote the evolutionary maintenance of PXI. This hypothesis is plausible, given the pronounced sexual size dimorphism in marsupials relative to other mammalian species (*e.g.*, [88], [89]). Such dimorphism is indicative of strong sexual selection, which could promote the accumulation of sexually antagonistic genetic variation and thereby limit opportunities to evolve RXI.

Once RXI has evolved, evolutionary reversals to strict PXI should face severe evolutionary constraints. In populations with RXI, the filtering of genetic variation by selection in females will strongly depend upon dominance. RXI permits the preferential accumulation of recessive, female-deleterious mutations, because such alleles experience weakened purifying selection. The retention of recessive alleles in populations with RXI should downwardly shift the mean dominance of segregating alleles (relative to the dominance coefficients of spontaneous mutations; *e.g.*, [12], [90]), and increase the cost to females of becoming homozygous or effectively haploid, as they would under PXI. Filtering of mutations based on their dominance coefficients does not eliminate opportunities to evolve biased RXI, but it should severely constrain evolutionary transitions to complete PXI, which completely eliminates effects of masking. This situation is analogous to the coevolution of outcrossing rates and inbreeding depression, with the latter expected to become more severe in outbreeding populations because they shelter recessive alleles from natural selection [91], [92].

### Species diversity for X inactivation strategies

Species-specific properties of mutation and genetic variation might predictably affect patterns of selection for different X inactivation strategies. Sexual selection and sex-biased mutation rates are each likely to vary among species, and both processes can influence the relative transmission probabilities of female-deleterious alleles between maternally and paternally derived X chromosomes.

Mammalian mutation rates are often higher in males than females [93], [94], which tends to upwardly bias paternal transmission of deleterious alleles. Consider a population with unbiased RXI and a mutation rate of *u_m_* and *u_f_* in males and females, respectively (see Text S1). With unbiased mutation (*u_m_* = *u_f_*), selection can favor reduced expression of the maternally inherited X when *s_f_h*<*s_m_*, as implied by eq. (8). Male-biased mutation (*u_m_*/*u_f_*>1) reduces this parameter space to (*u_m_*/*u_f_*)*s_f_h*<*s_m_*, because males transmit a higher fraction of de-novo mutations to their daughters. We predict that preferential inactivation of the paternally inherited X will be more common (or more severe) in species with strongly male-biased mutation rates.

Sexual selection could similarly favor paternally biased X inactivation. Although strong purifying selection in males via sexual selection can reduce paternal transmission rates of deleterious alleles ([33], [35], [36]; though not all data support this possibility, *e.g.*: [95], [96]), it will also reduce the frequencies and contributions of deleterious alleles to fitness variation in females. Sexual selection may simultaneously increase the pervasiveness of sexual antagonism [97] and the contribution of sexually antagonistic alleles to female fitness variation. If sexually antagonistic fitness variation increases with the strength of sexual selection, then so should the degree of paternally biased X inactivation.

## Methods

### X-linked modifier model

#### Haplotype frequency recursions

In a given generation, let the haplotype frequencies in eggs be *x*_1_ = [*A*_1_ *B*_1_], *x*_2_ = [*A*_2_ *B*_1_], *x*_3_ = [*A*_1_ *B*_2_], and *x*_4_ = [*A*_2_ *B*_2_]. Haplotype frequencies in sperm are *y*_1_ = [*A*_1_ *B*_1_], *y*_2_ = [*A*_2_ *B*_1_], *y*_3_ = [*A*_1_ *B*_2_], and *y*_4_ = [*A*_2_ *B*_2_]. Following random mating, females of the next generation will carry a maternally inherited haplotype *i* = {1, 2, 3, 4} and paternally inherited haplotype *j* = {1, 2, 3, 4} with probability *x_i_y_j_*. Males of the next generation inherit a single haplotype *i* from their mothers, with probability *x_i_*. The fitness of a female carrying haplotypes *i* and *j* is *f_ij_*, and the fitness of males carrying haplotype *i* is *m_i_*. Haplotype frequencies after selection and recombination, but prior to mutation, are described by the following set of recursion equations:
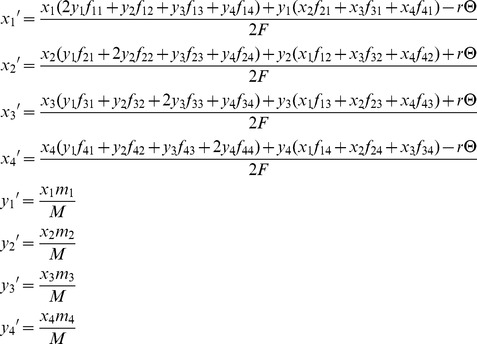
(10)where *F* is the sum of the numerators of *x*_1_′, *x*_2_′, *x*_3_′, and *x*_4_′; *M* is the sum of numerators of *y*_1_′, *y*_2_′, *y*_3_′, and *y*_4_′; and Θ = *x*_1_ *y*_4_ *f*_14_−*x*_2_ *y*_3_ *f*_23_−*x*_3_ *y*_2_ *f*_32_+*x*_4_ *y*_1_ *f*_41_.

For the mutation-selection balance scenario, we consider fitness variation contributed by alleles that are deleterious to both sexes, and arbitrarily assume that *A*_1_ is the deleterious allele. Assuming mutation rates at locus *A* are much smaller than the strength of selection against *A*_1_, mutations from *A*_1_ to *A*_2_ can be safely ignored. The frequency of each haplotype after a single generation, including mutation, will be:
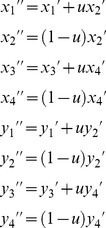
(11)where *u* is the mutation rate at the *A* locus, per gamete. When variation is maintained by sexual antagonism (that is, a balanced polymorphism is stably maintained), recurrent mutation will marginally affect equilibrium allele frequencies at the *A* locus [55]. Therefore, the recursion equations from eq. (10) are sufficient to describe haplotype frequency dynamics under sexual antagonism.

#### Invasion conditions for B_2_ alleles

Jacobian matrices were calculated for the two models of genetic variation for fitness (see Text S2), with each model giving rise to a block triangular matrix with two diagonal submatrices (see [46], chapter 12). In each model, invasion of the *B*_2_ allele is favored when the leading eigenvalue of the Jacobian is greater than one; *B*_2_ cannot invade when the leading eigenvalue is less than one. One submatrix has leading eigenvalue less than one as a condition of the population initially being fixed for *B*_1_ and at stable equilibrium at the *A* locus. Stability at *B* is therefore determined by the leading eigenvalue of the remaining submatrix, *J* (see Table S1 and Text S2).

Under the mutation-selection balance model for fitness variation, the characteristic polynomial is:
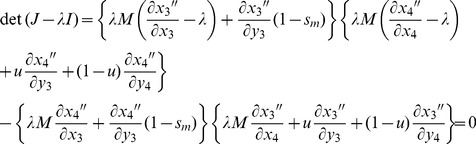
(12)where the partial derivatives are evaluated from the recursions in eq. (11), each evaluated at the equilibrium: 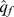 = *x*_1_ = 1−*x*_2_, 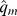 = *y*_1_ = 1−*y*_2_, and *x*_3_ = *x*_4_ = *y*_3_ = *y*_4_ = 0, with values of 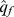 and 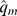 based on eq. (1). The leading eigenvalue is the largest of the roots of *λ*. Under the sexual antagonism model, the characteristic polynomial is:
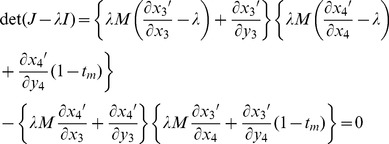
(13)where partial derivatives are calculated from recursions in eq. (10), each evaluated at the equilibrium: 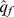 = *x*_1_ = 1−*x*_2_, 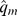 = *y*_1_ = 1−*y*_2_, and *x*_3_ = *x*_4_ = *y*_3_ = *y*_4_ = 0, with values of 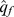 and 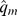 based on eq. (2).

Stability criteria (*i.e.*, whether the leading eigenvalue was greater or less than zero) were determined by hand, and leading eigenvalues were obtained numerically by Newton-Raphson iteration.

### Autosomal modifier model

When the modifier locus, *B*, is linked to an autosome, the haplotype recursions can again be obtained using similar approaches as described above. Here, the frequency of each haplotype in females will be the same as described above for the specific case of free recombination: *r* = ½. Haplotype frequencies in males, following selection and meiosis, are modified to:
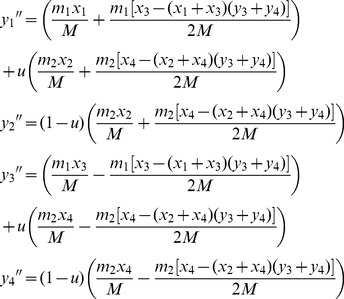
(14)where *m*_1_ = *m*_3_, *m*_2_ = *m*_4_, and *M* = (*x*_1_+*x*_3_)*m*_1_+(*x*_2_+*x*_4_)*m*_2_.

Stability analysis (invasion opportunities for rare *B*_2_ alleles) follows the same approach as before, with initial conditions of variation at locus *A* remaining unchanged. Under the case of variation maintained by sexually antagonistic selection, effects of mutation are ignored (we set *u* to zero). The generic characteristic polynomial is:
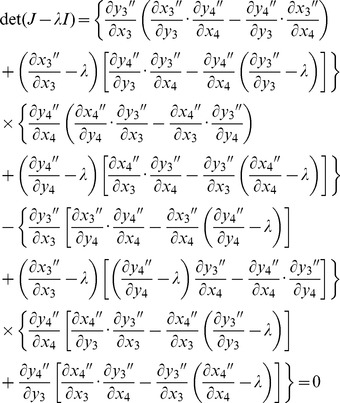
(15)with the partial derivatives each evaluated at the equilibrium: 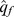 = *x*_1_ = 1−*x*_2_, 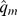 = *y*_1_ = 1−*y*_2_, and *x*_3_ = *x*_4_ = *y*_3_ = *y*_4_ = 0. Values of 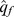 and 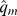 are based on eqs. (1) or (2) as appropriate.

## Supporting Information

Figure S1 The relative strength of selection on a modifier allele. This example plots the strength of selection for preferential inactivation of the maternal X (imposed by segregating deleterious alleles) relative to selection for a paternal X inactivation bias (imposed by sexually antagonistic alleles). In both cases, the modifier locus is linked to an autosome. A locus under purifying selection imposes selection on a modifier of strength *s*(del) = *λ_del_*−1, where *λ_del_* is the leading eigenvalue at the equilibrium with *B*_1_ fixed and *A*_1_ at mutation-selection balance (eq. (1) from the main text, with parameters *s_m_* = *s_f_*, *ξ*_11_ = ½, *ξ*_12_ = ½−10^−3^, *h* = 0.25, and *u* = 10^−5^). A sexually antagonistic locus imposes selection on a modifier *s*(SA) = *λ_SA_*−1, where *λ_SA_* is the leading eigenvalue at the equilibrium with *B*_1_ fixed and *A*_1_ at deterministic balanced polymorphism (eq. (2) from the main text, with parameters *t_m_* = *s_f_*, *ξ*_11_ = ½, *ξ*_12_ = ½+10^−3^, *h* = 0.25). The *y*-axis plots the relative strength of selection imposed by the two types of fitness loci, *i.e.*, the ratio: *s*(del)/*s*(SA).
(TIF)

Table S1 Fitness for the two locus system.
(PDF)

Text S1 Polymorphism at the *A* locus.
(DOC)

Text S2 Two locus fitness and stability.
(DOC)
