# Supplementary figures and images for: Sex-Differential Selection and the Evolution of X Inactivation Strategies
Source: PLoS Genet. 2013 Apr 18;9(4):e1003440. doi: 10.1371/journal.pgen.1003440 (PMC3630082; doi:10.1371/journal.pgen.1003440)

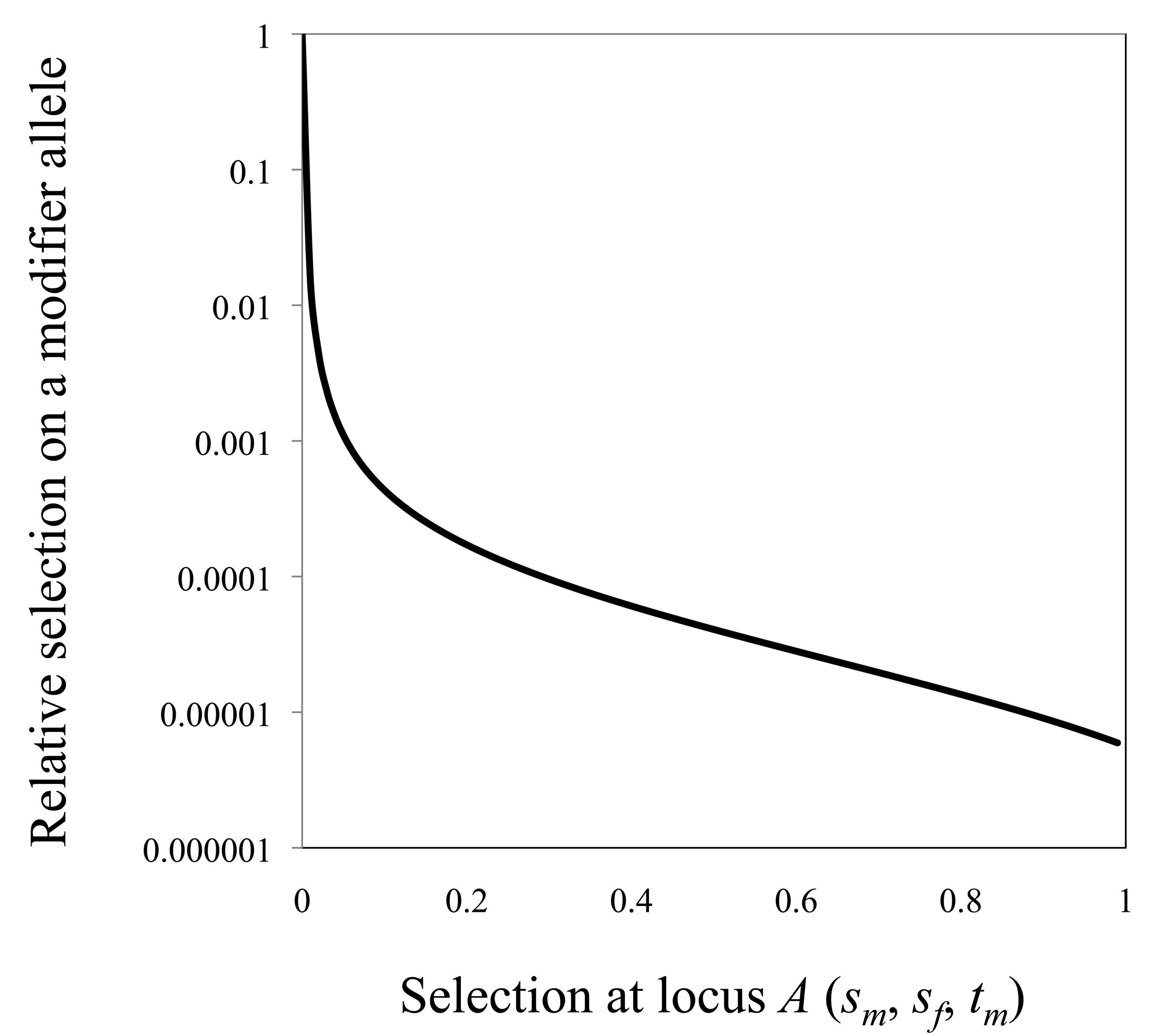

Supplement: Figure S1 — The relative strength of selection on a modifier allele. This example plots the strength of selection for preferential inactivation of the maternal X (imposed by segregating deleterious alleles) relative to selection for a paternal X inactivation bias (imposed by sexually antagonistic alleles). In both cases, the modifier locus is linked to an autosome. A locus under purifying selection imposes selection on a modifier of strength s(del) = λdel−1, where λdel is the leading eigenvalue at the equilibrium with B 1 fixed and A 1 at mutation-selection balance (eq. (1) from the main text, with parameters sm = sf, ξ 11 = ½, ξ 12 = ½−10−3, h = 0.25, and u = 10−5). A sexually antagonistic locus imposes selection on a modifier s(SA) = λSA−1, where λSA is the leading eigenvalue at the equilibrium with B 1 fixed and A 1 at deterministic balanced polymorphism (eq. (2) from the main text, with parameters tm = sf, ξ 11 = ½, ξ 12 = ½+10−3, h = 0.25). The y-axis plots the relative strength of selection imposed by the two types of fitness loci, i.e., the ratio: s(del)/s(SA). (TIF) [file pgen.1003440.s001.tif]
