# Supplementary material for: Sex-Differential Selection and the Evolution of X Inactivation Strategies
Source: PLoS Genet. 2013 Apr 18;9(4):e1003440. doi: 10.1371/journal.pgen.1003440 (PMC3630082; doi:10.1371/journal.pgen.1003440)
Supplement: Table S1 — Fitness for the two locus system. (PDF) [file pgen.1003440.s002.pdf]

Table S1

Table S1. Fitness for the two locus system

Female Fitness

Paternal haplotype	Maternal haplotype A_1B_1	A_2B_1	A_1B_2	A_2B_2
A_1B_1	$f_{11} = 1 - s_f$	$f_{21} = 1 - s_f(1 - \xi_{11})^k$	$f_{31} = 1 - s_f$	$f_{41} = 1 - s_f(1 - \xi_{12})^k$
A_2B_1	$f_{12} = 1 - s_f(\xi_{11})^k$	$f_{22} = 1$	$f_{32} = 1 - s_f(\xi_{12})^k$	$f_{42} = 1$
A_1B_2	$f_{13} = 1 - s_f$	$f_{23} = 1 - s_f(1 - \xi_{12})^k$	$f_{33} = 1 - s_f$	$f_{43} = 1 - s_f(1 - \xi_{22})^k$
A_2B_2	$f_{14} = 1 - s_f(\xi_{12})^k$	$f_{24} = 1$	$f_{34} = 1 - s_f(\xi_{22})^k$	$f_{44} = 1$

Male Fitness

A_1 deleterious	$m_1 = 1 - s_m$	$m_2 = 1$	$m_3 = 1 - s_m$	$m_4 = 1$
A_2 deleterious	$m_1 = 1$	$m_2 = 1 - t_m$	$m_3 = 1$	$m_4 = 1 - t_m$
