# Supplementary material for: Sex-Differential Selection and the Evolution of X Inactivation Strategies
Source: PLoS Genet. 2013 Apr 18;9(4):e1003440. doi: 10.1371/journal.pgen.1003440 (PMC3630082; doi:10.1371/journal.pgen.1003440)
Supplement: Text S1 — Polymorphism at the A locus. (DOC) [file pgen.1003440.s003.doc]

**Text S1. Polymorphism at the *A* locus**

**Deleterious mutation model.** Allele frequencies in males and females after selection are:

where $\bar{w}_{f}$ and $\bar{w}_{m}$ refer to mean female and male fitness with respect to this locus. The approximations are accurate near equilibrium as long as *A*_2_ is partially expressed in heterozygous females and/or in males, and the mutation rate is low. After mutation (at rate *u_m_* in males an *u_f_* in females), the frequency in gametes will be:

q*_f_*'' = q*_f_*'(1 – *u_f_*) + *u_f_*(1 – q*_f_*') ≈ *q_f_*' + *u_f_*

*q_m_*'' = *q_m_*'(1 – *u_m_*) + *u_m_*(1 – *q_m_*') ≈ *q_m_*' + *u_m_*

Solving for the equilibrium, *q_f_*'' = *q_f_* and *q_m_*'' = *q_m_*, leads to:

By substituting *u* = *u_m_* = *u_f_*, we obtain a result equivalent to that of Santure and Spencer (2012; p. 1460) [52], which is presented in eq. (1) of the main text.

**Sexual antagonism model.** Equilibrium frequencies under sexually antagonistic balancing selection have been derived previously by Seymour and Pomiankowski (2006) [43], Patten and Haig (2009) [54], and Santure and Spencer (2012) [52]. For our fitness parameterization (Table 1), and assuming that there is balancing selection at the locus, equilibrium frequencies of *A*_1_ are:

presented as eq. (2) in the main text.

Under balancing selection, 0 < $\hat{q}_{f}$ < 1, which is true under the criteria:

Several special cases of *h_pat_* and *h_mat_* lead to previously published results for sexually antagonistic polymorphism. For example, when the paternally inherited X is ubiquitously silenced (*h_mat_* = 1; *h_pat_* = 0), females and males are functionally haploid for the X, and the criteria for balancing selection is *s_f_*/(1 + *s_f_*) < *t_m_* < *s_f_*/(1 –*s_f_*). This result is identical to the criteria for autosomal, additive inheritance (Kidwell et al. 1977 [39]). For the case of complete maternal X inactivation (*h_mat_* = 0; *h_pat_* = 1), polymorphism can never be obtained (the criteria becomes: *s_f_* < *t_m_* < *s_f_*), as recently reported by Santure and Spencer (2012) [52]. For unbiased RXI (*h_mat_* = *h_pat_* = *h*), the criteria is 2*s_f_h*/(1 + *s_f_h*) < *t_m_* < 2*s_f_*(1 – *h*)/(1 – *s_f_h*), as reported in Patten and Haig (2009) [54]. Finally, for the additive case (*h* = ½), we get *s_f_*/(1 + *s_f_*/2) < *t_m_* < *s_f_*/(1 – *s_f_*/2), as reported in Pamilo (1979).

**Additional Reference:**

Pamilo P (1979) Genic variation at sex-linked loci: quantification of regular selection models. Hereditas 91:129-133.
