# Supplementary material for: Sex-Differential Selection and the Evolution of X Inactivation Strategies
Source: PLoS Genet. 2013 Apr 18;9(4):e1003440. doi: 10.1371/journal.pgen.1003440 (PMC3630082; doi:10.1371/journal.pgen.1003440)
Supplement: Text S2 — Two locus fitness and stability. (DOC) [file pgen.1003440.s004.doc]

**Text S2. Two Locus Fitness and Stability**

***Fitness Parameterization of the Two-Locus System.*** For the three *B* locus genotypes, female X inactivation rules are: *ξ*_11_ for *B*_1_*B*_1_, *ξ*_12_ for *B*_1_*B*_2_, and *ξ*_22_ for *B*_2_*B*_2_. Given the dominance functions outlined in the main manuscript, the fitness parameterization for the two-locus system is defined in Table S1.

***Invasion Criteria for a Modifier Allele.*** Invasion of derived *B*_2_ alleles are determined by stability analysis of the equilibrium with *x*_3_ = *y*_3_ = *x*_4_ = *y*_4_ = 0 (*i.e.*, *B*_1_ fixed in the population), which is determined by the leading eigenvalue of the following Jacobian matrix:

Under the deleterious mutation model of fitness variation (with *u* > 0), the matrix evaluates to:

Under sexually antagonistic balancing selection (with *u* = 0; see the main text), the matrix evaluates to:
